# Application and curative effect of laparoscopic purse-string sutures in the treatment of adult acute complicated appendicitis

**DOI:** 10.1186/s12893-022-01884-6

**Published:** 2023-01-04

**Authors:** Wenzhong Bao, Jie Wang, Dawei Tang, Liang Li, Xiangling Meng

**Affiliations:** 1 Department of Surgery, The Second People’s Hospital of Hefei, Hefei Hospital Affiliated to Anhui Medical University, Guangde Road, Yaohai District, Hefei, 230011 Anhui China; 2 Department of Surgery, The First Affiliated Hospital Anhui Medical University, Hefei, 230031 Anhui China

**Keywords:** Abdominal infection, Complicated appendicitis, Laparoscopy, Laparoscopic purse-string suture, Simple Hem-o-lok^®^ clamp, Stump closure

## Abstract

**Objective:**

To investigate the effect of laparoscopic purse-string sutures in adult complicated appendicitis treatment.

**Methods:**

The data of 568 adult cases of complicated appendicitis treated by laparoscopic appendectomy at the Hefei Second People’s Hospital, Anhui Province, China, from September 2018 to September 2021 were analysed retrospectively. The patients were divided into two groups: 295 cases in the laparoscopic purse-string suture treatment group (observation group) and 273 cases in the simple Hem-o-lok^®^ clamp treatment group (control group). The baseline data collected included age, gender, preoperative body temperature, leukocyte count and percentage of neutrophils and the surgery time. The postoperative data collected included antibiotic treatment duration, drainage tube placement time and the incidence of complications.

**Results:**

There were no significant differences in the baseline data of the two groups, including age, gender, preoperative body temperature, leukocyte count and neutrophil percentage (all *P* > 0.05). Compared with the control group, the postoperative hospital length of stay, duration of antibiotic treatment, the recovery time of peripheral white blood cell and neutrophil counts and the incidence of postoperative complications in the observation group were significantly decreased (*P* < 0.05).

**Conclusion:**

Purse-string sutures can effectively reduce the incidence of postoperative complications after a laparoscopic appendectomy for adult acute complicated appendicitis. There was faster postoperative recovery when patients’ appendiceal stumps were treated with laparoscopic purse-string sutures.

## Introduction

The development and improvement of laparoscopic techniques and instruments mean that most laparoscopic operations, including radical appendectomy, gastrectomy and hepatic lobectomy, can be performed. Acute appendicitis is the most common acute abdomen in abdominal surgery. The key to successful appendicitis surgery is the reliable management of the appendix stump and adequate and unobstructed drainage of the peritoneal exudates. Traditional laparoscopic appendectomy is usually performed with stump ligation or the Hem-o-lok^®^ clamp, which may result in poor ligation, looseness and cutting injuries, followed by intestinal flora translocation. Furthermore, the appendiceal stump of the mini fistula caused by abdominal infection occurs from time to time [1, 2]. Therefore, reducing the incidence of abdominal infection after laparoscopic appendectomy is a reality that surgeons face. Although laparoscopic purse-string suture to the serosal appendiceal stump can effectively avoid the problems of weak ligation and loose detachment due to the simple Hem-o-lok clamp [3], whether it can effectively reduce the incidence of postoperative abdominal infection is presently unknown. In addition, there is no definite conclusion about the operation mode in complicated acute appendicitis surgery. In this study, 568 adult cases of acute complicated appendicitis were analysed retrospectively after emergency laparoscopic appendectomy to explore the safety and efficacy of the procedure. Furthermore, this study aims to provide a reference for the follow-up clinical treatment of appendicitis.

## Data and methods

### Subjects

A retrospective cohort study was conducted to collect the clinical data of patients admitted to the General Surgery Department of the Second People’s Hospital of Hefei, Anhui Province, China, for acute appendicitis. The patients had all undergone laparoscopic appendectomy between September 2018 and September 2021. The inclusion criteria were as follows: (1) age > 18 years; (2) acute suppurative or gangrenous appendicitis with perforated appendix and localised or diffuse peritonitis; and (3) appendicitis with periappendiceal abscess formation, confirmed by postoperative pathology. The exclusion criteria for patients were as follows: (1) an appendiceal or ileocecal tumour diagnosis by postoperative pathology; (2) an appendectomy combined with other surgery; (3) acute simple appendicitis, chronic appendicitis or perforation of the appendix root that could not be ligated; and (4) conversion to open surgery. This study was approved by the Ethics Committee of the Second People’s Hospital of Hefei.

### Definition of complicated appendicitis in adults

Complicated appendicitis is not an exact anatomical concept, and there is presently no standard definition [4]. Rather, it is a clinical concept limited to surgeons. Complicated appendicitis is the opposite of simple appendicitis. Clinical studies [5, 6] have shown that most acute uncomplicated appendicitis can be cured by antibiotic treatment. However, distinguishing between simple and complicated appendicitis in the absence of surgery is a hot topic in current clinical research. By statistical analysis of preoperative computerised tomography (CT) scans [7] of acute appendicitis [8], the volume and distribution of platelets in the blood have been reported. The levels of bilirubin [9], serum sodium [10] and 5-hydroxyindoleacetic acid [11] and the neutrophil to lymphocyte ratio [12, 13] have been used to distinguish between simple and complicated appendicitis. However, the specificity was not high, and pathology or intraoperative exploration remains the gold standard for simple or complicated appendicitis diagnosis. This study evaluated suppurative, phlegmonous and catarrhal appendicitis as simple appendicitis and gangrenous, plastron and perforated appendicitis as complicated appendicitis.

### Surgical methods

Three skilled and experienced attending surgeons performed laparoscopic appendectomies on patients with complicated and simple appendicitis; all patients were treated following a similar surgery protocol. A navel hole of 10 mm was made as the cavity mirror observation hole; a 5 mm trocar was placed about 1 cm below the intersection of the bilateral anterior superior iliac spine and right middle clavicle as an auxiliary operating hole. At the bilateral anterior superior iliac spine level, a 10 mm trocar was placed at the 1–2 cm lateral edge of the left rectus abdominis intersect point; this was used as the main operating hole. The inferior epigastric artery should be avoided to prevent iatrogenic injury during the puncture of the operating holes using a laparoscope. After the appendectomy, a swab bag was placed and removed from the left main operating hole; the abdominal drainage tube was withdrawn from the right trocar hole.

The mesentery of the appendix was clamped with a Hem-o-lok clamp and separated to the root after the appendectomy in the laparoscopic purse-string suture group (observation group); the appendix was ligated with silk thread 5 mm from the base of the appendix and then closed with a 3–0 absorbable barbed thread. In the simple Hem-o-lok group (control group), the mesentery of the appendix was also closed with a Hem-o-lok by directly double-clamping the roots. After aspirating the pus, local use was made of a small gauze to wipe clean, not associated with diffuse peritonitis when not to flush, after the routine placement of a drainage tube from the right trocar hole.

### Observable indicators

General information about the patients was collected before surgery; this included age, sex, preoperative body temperature, peripheral white blood cell (WBC) count and the percentage of neutrophils. The operative time was recorded, and the following postoperative information was collected: duration of hospital stay, fluid diet, antibiotic use, drainage tube placement, the recovery time of peripheral WBC neutrophil count and complications, including abdominal and incision infections.

The diagnostic criteria for abdominal infection were as follows: (1) fever over 38.4 °C, accompanied by abdominal pain and distention, rectal irritation and a continuous increase of the WBC; (2) a painful mass in the anterior wall of the rectum diagnosed by digital rectal examination; and (3) a CT scan of the abdomen after surgery that showed encapsulated fluid or abscess formation in the ileocecal region of the right lower abdomen; infections other than intraperitoneal infections were excluded [14].

According to the diagnostic criteria of nosocomial infections, the following were considered: (1) superficial incision infection with redness, swelling, heat, pain or purulent discharge or clinical diagnosis; (2) drainage from deep incision or aspiration of pus (except for drainage after infectious operation), (3) surgical or spontaneous wound dehiscence, purulent secretion, fever ≥ 38 °C or local pain or tenderness; (4) evidence of an abscess or other infection involving a deep incision found by reoperation, histopathology or imaging examination [15].

If the amount of drained ascites was less than 20 mL/day, then the drainage tube can be removed. The absence of abdominal and pelvic effusions was confirmed by ileocecal imaging using a colour Doppler ultrasound or CT scan 3–5 days after surgery [16].

Since the bacteria of acute appendicitis infection are mostly *Escherichia coli* [17], patients were treated with a third-generation cephalosporin antibiotic after surgery. All patients received the same antibiotic treatment. The criteria for withdrawal of antibiotics were the recovery of a normal diet, body temperature and peripheral WBC and neutrophil count.

### Statistical methods

SPSS^®^ (Statistical Software™ Version 21.0, IBM SPSS Statistics, IL, USA) was used for statistical analysis. The Kolmogorov–Smirnov test was used to test the normality of the measured data. The measurement data were described statistically by x ± s; the comparison between groups was performed by an independent sample *t*-test. Count data were statistically described as cases (%), and Fisher’s precision probability test was used to compare the groups; *P* < 0.05 indicated statistical significance.

## Results

### Comparison of general preoperative information between the two groups

A total of 568 adult patients with acute complicated appendicitis were included in this study, including 295 patients in the laparoscopic purse-string suture group (observation group) and 273 cases in the simple Hem-o-lok group (control group). The general data of the two groups were analysed and compared. No significant differences in age, sex, preoperative temperature, WBC count, neutrophil percentage and pathological types were seen between the two groups (*P* > 0.05). Therefore, it was confirmed that the baseline data of the two groups were comparable (see Table 1).

**Table 1:** Comparison of general data of patients with complex acute appendicitis between the two groups [n (%)]

Variables	Observation group (n = 295)	Control Group (n = 273)	t/χ^2^ value	*P* value
Age (year, $\overline{{\text{X}}}$ ± s)	41.5 ± 11.7	42.8 ± 12.2	1.296	0.195
Male/female	198/97	179/94	0.153	0.696
Body temperature (℃, $\overline{{\text{X}}}$ ± s)	37.9 ± 1.1	37.8 ± 1.0	1.131	0.259
Peripheral white blood cells (× 10^9^ $\overline{{\text{X}}}$ ± s)	13.4 ± 3.9	13.7 ± 4.1	0.894	0.372
Peripheral blood neutrophil(10^9^, $\overline{{\text{X}}}$ ± s)	82.8 ± 9.7	83.6 ± 10.2	0.958	0.338
Type of pathological diagnosis				
Purulent	234 (79.3)	217 (79.5)	0.002	0.961
Gangrene	61 (20.7)	56 (20.5)		
Complex types				
Combined intestinal dilatation	22 (7.5)	26 (9.5)	0.781	0.377
Combined appendiceal perforation	14 (4.7)	17 (6.2)	0.602	0.474
Combined appendiceal abscess	6 (2.0)	7 (2.6)	0.178	0.673
Retroperitoneal appendix	4 (1.4)	4 (1.5)	0.006	0.938

### Comparison of intraoperative and postoperative indexes

There was no significant difference in the operating time (50.1 ± 15.1 min vs 47.7 ± 4.8 min) and the time of the first meal (36.1 ± 11.7 h vs 35.4 ± 11.6 h) between the two groups (*P* > 0.05).

The postoperative length of hospital stay was shorter in the observation group (5.8 1.2 days) compared to the control group (6.1 ± 1.3 days) (*P* < 0.05). The duration of antibiotic use in the observation group was (4.7 ± 0.4 days), which was significantly lower than that in the control group (4.9 ± 0.5 days) (*P* < 0.05). The recovery time of the peripheral WBC count (4.4 ± 0.4 vs 4.7 ± 0.4 days) and the recovery time of the neutrophil count (4.5 ± 0.4 days vs 4.7 ± 0.5 days) in the observation group was shorter than that in the control group; the difference was statistically significant (*P* < 0.05). The incidence of postoperative complications in the observation group was 7.12%, which was significantly lower than in the control group (15.02%) (*P* < 0.05). Moreover, the total operation cost in the observation group (10,836.6 ± 115.4 RMB) was significantly lower than that in the control group (10,908.8 ± 122.6 RMB); the difference was statistically significant (*P* < 0.05) (see Table 2).

**Table 2:** Comparison of postoperative parameters in patients with complicated acute appendicitis between the two groups [n (%)]

Index	Control Group(n = 273)	Observation group(n = 295)	t/χ^2^ value	*P* value
Operation time (min, $\overline{{\text{X}}}$ ± s)	47.7 ± 14.8	50.1 ± 15.1	1.912	0.061
Time of first meal after operation(h, $\overline{{\text{X}}}$ ± s)	35.4 ± 11.6	36.1 ± 11.7	0.716	0.475
Postoperative hospital length of stay(d, $\overline{{\text{X}}}$ ± s)	6.1 ± 1.3	5.8 ± 1.2	2.860	0.004
Duration of antibiotic use(d, $\overline{{\text{X}}}$ ± s)	4.9 ± 0.5	4.7 ± 0.4	5.282	< 0.001
Postoperative drainage tube placement time(h, $\overline{{\text{X}}}$ ± s)	73.7 ± 5.4	72.9 ± 5.3	1.780	0.076
The recovery time of peripheral white blood cell count (d)	4.7 ± 0.4	4.4 ± 0.4	8.931	< 0.001
Recovery time of neutrophil count (D)	4.7 ± 0.5	4.5 ± 0.4	5.282	< 0.001
Postoperative complications	41 (15.02%)	21 (7.12%)	9.099	0.003
Residual infection of abdominal cavity	8	1		
Incision infection	1	1		
Intestinal obstruction	32	19		
Total operating cost (yuan)	10,908.8 ± 122.6	10,836.6 ± 115.4	7.241	0.00

## Discussion

Acute appendicitis commonly results in abdominal surgery, although clinical studies report that acute simple appendicitis can be cured by intravenous or oral administration of antibiotics. However, surgical treatment is still the first choice for the treatment of complicated appendicitis [5, 6]. Studies have shown that older patients [18, 19] benefit more from a laparoscopic than an open appendectomy, including benefits such as smaller surgical wounds and shorter hospital stays. However, due to the serious oedema of local tissues, variation of the anatomical structures and doctors’ different proficiencies, acute complicated appendectomies are inevitably transferred to open surgery. Moreover, the conversion to a laparotomy for complicated appendices has been reported clinically to only be between 4.3% and 9.7% [20, 21].

Incision infection and abdominal cavity infection are common complications of acute appendicitis after open or laparoscopic appendectomy. Furthermore, the occurrence of intra-abdominal infection is reported to be closely related to the effectiveness of root treatment, the unobstructed drainage of intra-abdominal purulent exudate and the rational use of antibiotics. Clinical reports [22] suggest no difference in the incidence of intra-abdominal infections after laparoscopic or open appendectomies for either simple or complicated appendicitis. However, statistical evidence [23] supports that the incidence of incision infection after laparoscopic appendectomy is reduced, but the incidence of abdominal infection is higher. This study showed that a laparoscopic purse-string suture is associated with a lower incidence of postoperative intraperitoneal or incisional infection; this statistical difference may be related to differences in the selected sample. Additionally, for a laparoscopic appendectomy, the higher incidence of intra-abdominal infection after a complicated appendectomy [24], may be related to the translocation of intestinal flora and transmural infection caused by the unsatisfactory management of the appendiceal root.

Retrospective study statistics do not indicate an association between postoperative drainage and the occurrence of abdominal infection in patients with complicated appendices, regardless of laparotomy [25] or laparoscopy [26]. Furthermore, studies [27] suggest that the duration of broad-spectrum antibiotic treatment after an acute and complicated appendectomy is not associated with the development of intra-abdominal infections. Previous studies have found that using antibiotics for intra-abdominal space infections after a laparoscopic appendectomy in children does not shorten the cure time [28] and may even lead to adverse effects from the long-term use of antibiotics. However, the duration of hospital stay, the duration of antibiotic use, the recovery time of peripheral WBC and neutrophil counts was significantly shorter in the study group than in the control group. The reasons for the inconsistent results may include sampling errors and different criteria for judging postoperative abdominal infection. It is suggested that many factors cause abdominal infection after an appendectomy; the key to preventing infection is dealing with the root of the appendix satisfactorily, effectively draining the abdominal cavity and using broad-spectrum antibiotics postoperatively.

Traditional open appendectomy usually uses purse-string sutures, while Laparoscopic appendectomy (LA) is mainly performed with ligators, Hem-o-lok clamps, or cutting closures; there are also [29] cases reported where only LigaSure™ technology has been used to close the appendix stump. Hem-o-lok is primarily used for duct-like structures, such as blood vessels and bile ducts; the appendix, although duct-like, is an intestinal structure and tissue oedema is evident during acute inflammation. The Hem-o-lok has a strong cutting force and may not show morphological changes in a short period. Still, the cutting injury may lead to a damaging inflammation caused by the rupture of the muscle fibres in the appendix wall; in severe cases, the mucosa can be injured, and the intestinal flora transferred to cause abdominal infection. Studies have shown that the incidence of abdominal infection after laparoscopic appendectomy with a cutting closure device is high; if the appendix stump is double ligated with a ligator device and the distance between the two ligature lines is more than 2 mm, the incidence of postoperative abdominal infection may be increased [30]. No difference was reported in the incidence of intra-abdominal infection after appendiceal stump ligation with a [31] ligator (that is, simple silk ligation of the appendiceal stump) compared with a Hem-o-lok clamp alone. Complicated appendicitis surgery, whether using a double ligature or a Hem-o-lok double clamp, may result in the translocation of intestinal flora due to inflammation and mucosal damage, which may cause postoperative abdominal infection. Antibiotic treatment discontinuation and drainage tube removal after a laparoscopic appendectomy is mainly judged by clinical symptoms, laboratory tests and imaging, including no abdominal pain or fever, a return to normal WBC and neutrophil counts and no ascites on imaging. This study showed no significant difference between the two groups (*P* > 0.05). Furthermore, the results suggested that using a purse-string suture can reduce local oedematous of the serosa tissue, avoid inflammation and abdominal abscesses caused by the cut injury of the Hem-o-lok clamp; this is beneficial to the patient’s rapid recovery.

In summary, the authors of this study performed two procedures in both groups. The use of purse-string embedding was statistically superior to the Hem-o-lok clamp in the treatment of adult acute complicated appendices. However, there were inadequacies. First, this study was a retrospective statistical analysis. Despite the inclusion of strict exclusion criteria, statistical errors caused by selective bias are inevitable. Second, this study’s presupposition is that a laparoscopic appendectomy with purse-string sutures is a skilful technique, which may lead to different postoperative complications in patients. Therefore, further prospective controlled studies are needed to test the effectiveness of purse-string sutures for this application.

## Conclusion

In conclusion, a laparoscopic appendectomy combined with purse-string closure is more effective than using a Hem-o-lok closure in reducing the incidence of postoperative complications, reducing the use of antibiotics and shortening the length of hospital stays. Furthermore, it is beneficial to the rapid recovery of patients.

## Data Availability

All data generated or analyzed during this study are included in this article.
